# Relevance of Soil Heavy Metal XRF Screening for Quality and Landscaping of Public Playgrounds

**DOI:** 10.3390/toxics11060530

**Published:** 2023-06-14

**Authors:** Oana Răcușan Ghircoiaș, Claudiu Tănăselia, Mircea Chintoanu, Ioana Crișan, Adela Hoble, Răzvan Ștefan, Marcel Dîrja

**Affiliations:** 1 Department of Earth Survey and Exact Sciences, Faculty of Forestry and Cadaster, University of Agricultural Sciences and Veterinary Medicine of Cluj-Napoca, Calea Mănăștur Street No. 3–5, 400372 Cluj-Napoca, Romania; oana.ghircoias@usamvcluj.ro (O.R.G.); marcel.dirja@usamvcluj.ro (M.D.); 2 Institute for Analytical Instrumentation Subsidiary of National Institute of Research and Development for Optoelectronics INOE 2000 Donath Street No. 67, 400293 Cluj-Napoca, Romania; claudiu.tanaselia@icia.ro (C.T.); mircea.chintoanu@icia.ro (M.C.); 3 Department of Crop Science, Faculty of Agriculture, University of Agricultural Sciences and Veterinary Medicine of Cluj-Napoca, Calea Mănăștur Street No. 3–5, 400372 Cluj-Napoca, Romania; 4 Department of Horticulture and Landscaping, Faculty of Horticulture and Business in Rural Development, University of Agricultural Sciences and Veterinary Medicine of Cluj-Napoca, Calea Mănăștur Street No. 3–5, 400372 Cluj-Napoca, Romania; adela.hoble@usamvcluj.ro; 5 Department of Physics, Faculty of Veterinary Medicine, University of Agricultural Sciences and Veterinary Medicine of Cluj-Napoca, Calea Mănăștur Street No. 3–5, 400372 Cluj-Napoca, Romania

**Keywords:** urban, pollutants, residential area, health, vegetation

## Abstract

Heavy metals have become widespread urban pollutants, exposing vulnerable age groups such as children to potential risk. Specialists need feasible approaches that can routinely assist them in customizing options for sustainable and safer urban playgrounds. The aim of this research was to explore the practical relevance of the X-ray Fluorescence (XRF) method from the perspective of landscaping specialists, and the practical significance of screening for those heavy metals that currently present elevated levels across urban environments Europe-wide. Soil samples from six public children’s playgrounds of different typologies from Cluj-Napoca, Romania, were analyzed. The results indicated that this method was sensitive to identifying thresholds stipulated in legislation for the screened elements (V, Cr, Mn, Ni, Cu, Zn, As, and Pb). Coupled with the calculation of pollution indexes, this method can serve as a quick orientation in landscaping options for urban playgrounds. The pollution load index (*PLI*) for the screened metals showed that three sites displayed baseline pollution with incipient deterioration in soil quality (*PLI* = 1.01–1.51). The highest contribution to the *PLI* among the screened elements, depending on the site, was due to Zn, Pb, As, and Mn. The average levels of the detected heavy metals were within admissible limits according to national legislation. Implementable protocols addressed to different categories of specialists could help to transition towards safer playgrounds and more research on accurate cost-effective procedures to overcome the limitations of existing approaches is currently needed.

## 1. Introduction

According to the United Nations, currently over half of the world’s population lives in urban areas and by 2030 more than 60% will live in cities [1]. Therefore, the quality of the urban environment is impacting the lives of billions of people. Concomitantly with rapid urbanization in recent decades, the greenness of cities worldwide increased by 12%, while in European cities, it rose by 38%. Currently, about 44% of the European urban population is living within 300 m of a public park, with variations across countries and regions [2]. This comes in response to the need for natural space to engage in outdoor recreative activities by the urban population, from children to the elderly [3]. While, on average, the European urban population enjoys around 18.2 m^2^ of publicly accessible green space per inhabitant, in Cluj-Napoca this is only 5.61 m^2^, lower than the country capital city of Bucharest, or many cities from Western Europe of similar size [2]. Urban parks and playgrounds are important parts of urban infrastructure [4]. Although these spaces are recognized for their conducive role to the positive overall well-being of urban inhabitants of all ages [5,6], they considerably benefit children [7]. It has been extensively shown that urban planning has to consider child-friendly spaces [8]. Urban playgrounds are important instruments that enable learning through the environment, and their availability can be linked to healthy child development [9]. As metropolitan areas continue to extend, and new playgrounds are created across residential areas, their quality should become a priority in order to ensure these are healthy and safe spaces. 

Increasing levels of heavy metals (HMs) in urban environments are concerning [10] due to the potential health impact and public green spaces are also under this pollution threat. In cities, frequently the atmospheric pollution creates discomfort for people, but metals accumulate in soil and, since they are not degraded, pose a risk to plants, animals, and humans. Therefore, long-term pollution is best evidenced in soils. Issues and concerns for the safety of children’s playgrounds usually revolve around the safety of play equipment that has to meet standards [11,12] and potential biologic contamination, particularly of sand pits [13]. Surprisingly, although parents and guardians have concerns about intense car traffic near the playgrounds, their concerns are not necessarily in regard to pollution [14], indicating insufficient awareness. 

HMs naturally occur in the lithosphere and soil [15], but HM accumulation in urban soil is a result of anthropogenic activity [16,17]. In urban soil, the accumulation of lead, zinc, cadmium, and copper is due to traffic and the construction materials used [18]. Additional sources of pollution are connected with households heating systems, and waste management [19,20], as well as the proximity to industrial and agricultural activity centers [20]. In post-industrial cities, passive pollution related to the historical past can also be present [21] and is most often represented by the persistence of heavy metals in soils [22], and their remanence levels are related to the scale and intensity of the former activities [23]. 

Most research on heavy metal contamination was conducted on agricultural soil. Thus, current knowledge of heavy metal pollution in recreational urban areas is limited [10]. Among the heavy metals, some have no known metabolic role in the human organism (cadmium, lead, and mercury) [24] and are toxic even in the smallest amounts [25]. Generally, HMs become dangerous only over a certain threshold, but some of them play a metabolic role and are essential for the organism in minute quantities (manganese, cobalt, copper, and zinc). For other elements, reports of potential metabolic roles were not clearly defined in the past and they were, therefore, often included in the non-essential group, but in light of later advances, they were proposed as possible essential trace elements: vanadium, chromium, nickel, and arsenic [26]. Exposure routes to heavy metals in humans are ingestion, inhalation, and dermal contact. In children, the health implications are reportedly more severe than in adults [27]. All systems of the body can be negatively affected [28] and this can result in a wide range of dysfunctions, from behavioral to neurocognitive disorders, respiratory to tumoral, and cardiovascular diseases [27]. 

By screening and monitoring environmental parameters, any degradation of quality can become subject to interventions with the purpose to restore the urban ecosystem to its optimal, safe state. 

There are several methods available for the detection and monitoring of heavy metal levels in soil, which are more or less laborious [29]. Studies on the levels of heavy metals in the soil in the urban environment in various regions of Europe were carried out, but there are still many gaps in knowledge [10]. Additionally, there is a persisting hurdle in going beyond reporting the levels and transitioning to routine practical approaches, but the reasons for this have received very little attention. Due to the permanent nature of the threat of heavy metals in the urban environment, quick and cheap methods are needed to assist landscaping specialists. A quick method of detection is XRF (X-ray fluorescence), which can provide fast results without laborious sample preparation protocols and for a wide range of environmental samples, including soil [30]. Additionally, useful indexes that could provide a measure of the threat and risk of HMs in soil have been proposed [31]. Out of the possible approaches, for landscaping architects the analytical method employed could be coarse but of sufficient sensibility for detecting at least normal limits and alert levels mentioned in legislation with an acceptable degree of accuracy. This could direct decisions in regard to the need to improve substrate quality, as well as customize vegetation options by choosing plant species tolerant to those heavy metals presenting elevated levels. 

The aim of this study was to explore the relevance of XRF screening for HMs from the perspective of the landscaping specialist, by screening for some of the most common heavy metals that present increased levels across European cities [10], in some public playgrounds in Cluj-Napoca, Romania. 

Four objectives were defined:

- Compare the measurements against threshold values from current legislation;
- The calculation of pollution indexes for the HMs measured to assess threat;
- An examination of the relationship between the identified heavy metals;
- Prospect the usefulness of XRF for landscaping approaches and identify limitations and challenges.

## 2. Materials and Methods

### 2.1. Location

Cluj-Napoca is the second-largest city in Romania, located in the hilly center of the historical region of Transylvania. The city represents an economic, cultural, and educational center that attracts young people from nearby regions who seek to establish themselves in the city. Out of the population of over 300,000, an important proportion is comprised of young people and children. The climate is temperate continental with four seasons [32]. 

This study was performed across six highly frequented children’s playgrounds located in Cluj-Napoca, Romania. The locations were residential areas with young families. Representative public-accessible playgrounds of different typologies were selected by considering: (1) different distances from the city center; (2) different covering vegetation types; and (3) different elevations (Table 1). 

The first two sites are located in the residential district closest to the city center, out of the six sites studied. The second two are located at higher elevations in the Bună Ziua-Zorilor area, a busy area but further from the city center compared to the first two. The last two locations are from the southeast of the city and, being the furthermost from the city center and located in the least polluted areas, away from intense traffic. Regarding the type of vegetation among study sites, there were playgrounds with lawn only, as well as with woody vegetation besides lawn. Because Cluj-Napoca is situated in the hilly valley of Someș River, different elevations were considered: CJ-G in the district of Gheogheni, in the vicinity of Becaș river, as the lowest point, while CJ-BZ1 and CJ-BZ2 in the residential area of highest elevation in the city.

### 2.2. Sampling and Chemical Analyses

At each sampling site, pH was measured with portable pH meter.

Soil samples were collected from top soil (0–10 cm) at each of the six sites in November 2022. Three independent samples per site were collected, resulting in 18 soil samples (6 × 3). Only top soil was collected for analysis because this presents the risk of dust lift-up, which could expose persons in the park and children whilst playing in the area. Samples were placed in plastic boxes and transported to the authorized laboratory at the “Research Institute for Analytical Instrumentation” in Cluj-Napoca for analysis. Three samples per site were analyzed using X-ray Fluorescence (XRF) technique. For XRF analysis, a Bruker Tracer 5i portable X-ray fluorescence instrument (Billerica, MA, USA) was used. All the readings were performed at normal atmospheric conditions (no vacuum, no helium flush). Quantitative results were obtained using the calibrated method for soil samples provided by the manufacturer. Data acquisition time interval for each sample was set at 60 s (with two 30 s runs, one optimized for low-Z elements and the other one optimized for high-Z elements). No filters were used for these measurements.

### 2.3. Data Interpretation and Statistical Analysis

The heavy metal measurements at each location were compared with threshold values from Romanian legislation in force [33].

For the identified heavy metals across the six sites, pollution indexes proposed in the literature were determined based on Formulas (1) and (2) [31].

Single pollution index (*PI*) was calculated based on the soil concentration for each heavy metal (*Cn*) at each site (1). This index shows which heavy metal poses risk out of those measured [31]. Geochemical background (*GB*) reference values were used for calculating *PI*. Geochemical background MAT11LU-6 values were used for heavy metals Cr, Ni, Cu, Zn, and Pb, according to the European Soil Database from the work of Utermann et al. [34]. The cited work does not show levels for As, V, and Mn. Therefore, for these measured elements (As, V, and Mn) we used as geochemical background levels the worldwide average values in silty-loamy soils given by Kabata-Pendias et al. [35]. Pollution load index (*PLI*) was further calculated as geometric average of *PI* (2). This index assesses the overall pollution in the soil at each site [31].

Single pollution index:

(1) $$PI=\frac{Cn}{GB}$$

Interpretation: class 1, *PI* < 1 soil = soil pollution absent; class 2, 1 < *PI* < 2 = low; class 3, 2 < *PI* < 3 = moderate; class 4, 3 < *PI* < 5 = strong; and class 5, *PI* > 5 = very strong [31].

Pollution load index:

(2) $$PLI=\sqrt[{n}]{PI1\times PI2\times \ldots PIn}$$

Interpretation on scale from 0 to 10: 0 < *PLI* ≤ 1—unpolluted; 1 < *PLI* ≤ 2—low; 2 < *PLI* ≤ 3—moderate; 3 < *PLI* ≤ 4—moderate to high; 4 < *PLI* ≤ 5—high; and *PLI* > 5—very high [36]. The values below 1 indicate no pollution present, those of 1 only baseline levels of pollution, and above 1 indicate some deterioration in soil quality.

The calculation of pollution indexes and percent difference from reference levels was conducted using Microsoft Excel (Microsoft, Washington, DC, USA).

Statistical analysis was conducted using PAST 4.0 (Natural History Museum, Oslo, Norway). Distribution of data was analyzed by Shapiro–Wilk test, which showed normal distribution assumption was met for V, Cr, Ni, and Pb, while normal distribution assumption was not met by Mn, Cu, Zn, and As data sets. Analysis of variance test was applied accordingly to identify the influence of site location on heavy metal measurements. When a significant difference between means was indicated by the analysis of variance, further post hoc test was applied to identify between which means the difference was significant. Relationships between variables were explored using Kendall tau, a distribution-free correlation test preferred for environmental data [37] and adopted in similar cases for exploring relationships related to various heavy metal levels in soil [38,39].

The analysis explored the potential and suitability of pollution indexes and comparison against reference threshold in obtaining quick orientation in landscaping concepts or interventions with the purpose to improve the quality of the playgrounds. Potential applicability of these approaches was further discussed based on the obtained parameters.

## 3. Results

### 3.1. Levels of Heavy Trace Elements

Out of the eight elements screened (V, Cr, Mn, Ni, Cu, Zn, As, and Pb), three elements were present in all eighteen soil samples analyzed (V, Mn, and Ni), while Cr was detected in less than half of the analyzed samples. Analysis of variance showed that the differences observed between the mean level of heavy metals among the six sites were statistically significant only for Zn concentration (Table 2). The coefficient of variation showed higher dispersion from the mean for Mn, suggesting the heterogenous presence of this element across the samples (Table 2).

In any landscaping design or intervention, the specific measurements registered at each given location are the most relevant ones, since any design plan is site-based (Table 3).

Average levels of heavy trace elements did not reach the intervention level stipulated in Romanian legislation for any of the detected elements (Table 3). However, the average As level at the site CJ-BZ1 reached alert levels compared to standard thresholds, and should be monitored. Apart from this, the others were below the alert level. Among the locations, CJ-AM1 had the highest Cu levels out of the ones investigated. CJ-AM2 had the highest levels of V, Ni, Zn, and Pb out of the screened locations. The site CJ-G was the only one where Cr was below levels of detection, and also had the lowest As levels out of the six. The site CJ-BZ2 had the highest levels of Mn but the lowest levels of V and Pb out of the six sites.

The reference levels for common heavy metals in Romanian top soil released by the National Research and Development Institute for Soil Science, Agrochemistry and Environment from Bucharest [40] were compared to the ones obtained in this study. It was observed that Mn levels were up to 56.27% higher (CJ-BZ2) or 70.36% lower (CJ-CB) compared to the national reference average in soil. Ni levels were between 24.90% higher (CJ-AM2) and 49.97% lower (CJ-BZ2), Cu levels were up to 49.02% higher (CJ-AM1) or 43.82% lower (CJ-BZ1), and Zn levels were 70.91% higher (CJ-AM2) or 37.89% lower (CJ-G). Pb levels were up to 109.36% higher (CJ-AM2) or 66.25% lower (CJ-BZ2) than the national average. As and V compared to the worldwide levels [35] were up to 47.96% (CJ-AM2) and 19.37% higher (CJ-BZ2), respectively. The Cr mean level was higher only by 5.18% (CJ-AM2) while for the other five locations, the levels were below the European background [34].

Thus, compared to reference values from the sources cited above, out of eight heavy metals measured, sites CJ-AM1 and CJ-AM2 both exceeded the mean reference values for seven out of eight heavy metals, site CJ-CB exceeded these in three out of eight, sites CJ-BZ1 and CJ-BZ2 for two out of eight, and CJ-G in only one out of eight. The only element that showed elevated levels compared to worldwide mean values at all studied locations was As. In addition, Pb showed elevated levels at four locations compared to Romanian mean levels. Thus, vegetation options should consider plant species that can tolerate and remediate these elements.

The single pollution indexes attributed to their corresponding pollution threat classes are presented in Table 4.

According to the interpretation, in four screened elements the indexes were within no-pollution to low pollution classes (V, Cr, Mn, and Ni). Apart from these, Cu poses a moderate threat at site CJ-AM1, Zn poses a moderate pollution threat at site CJ-AM1 and a strong pollution threat at site CJ-AM2, Pb poses a moderate pollution threat at sites CJ-AM1 and CJ-AM2, while As poses a moderate pollution threat at site CJ-BZ1 (Table 4).

Based on the results of the pollution load index, it was determined that site CJ-G had no pollution. Sites CJ-CB, CJ-BZ1, and CJ-BZ2 show only baseline pollution, while sites CJ-AM1 and CJ-AM2 show incipient or slight deterioration in soil quality.

For the studied children’s playgrounds, the highest contribution to the pollution load index of soil had the following elements, in descending order:

- CJ-AM1—Zn > Pb > Cu > As > Ni > Mn > V > Cr;
- CJ-AM2—Zn > Pb > Cu > As > Ni > V > Cr > Mn;
- CJ-BZ1—As > Pb > Zn > Ni > V > Cu > Mn > Cr;
- CJ-BZ2—Mn > As > Zn > Cu > Pb > V > Ni > Cr;
- CJ-CB—Pb > Zn > As > Ni > Cu > V > Cr > Mn;
- CJ-G—As > Pb > Zn > Ni > Cu > V > Mn.

It can be observed that sites CJ-AM1 and CJ-AM2 located within the same residential area (Andrei Mureșanu), which also have similar vegetation coverage (lawn and trees, delimited by hedges), had six out of the eight heavy metals with a similar contribution to the pollution load index, with Zn and Pb having the highest single pollution indexes. On more than two sides these two playgrounds have streets with intense car traffic, therefore the deposition from car exhaust pollution on surfaces and tree foliage with subsequent wash-off into the ground might be a source. In addition, the presence of concrete pathways at site CJ-AM1 and asphalt pathways at site CJ-AM2 might be potential sources of slow-release heavy metals with rain run-off. One can notice that at these two locations, the V levels were higher compared to the other four locations.

Sites CJ-BZ1 and CJ-BZ2 are situated at the highest elevation compared to the other locations (within Bună Ziua district), are covered only by lawn, and on two sides there are streets with intense car traffic. However, Zn is only third while Pb is second or fifth in the contribution to the pollution load index. Noticeably, as this area is situated at the highest elevation compared to the other areas of the city, these sites experience stronger air currents. Sites CJ-CB and CJ-G are not in the vicinity of roads, and are neighbored by vegetated areas. The first of these two is located in the Borhanci residential area (the furthest from the city center), where some neighboring lands are also used for gardening or agricultural activities. Therefore, for this site, the air drift of pesticides should be considered as an occasional or potential source of pollution. However, this site ranks within the baseline level, with low pollution. Site CJ-G is situated in Gheorgheni district, within a larger recreational and sports complex and is one of the most important for the city. It is situated at the lowest elevation and has the neighboring riparian vegetation of the Becaș river that flows nearby. This site was the “cleanest”, with no pollution. Therefore, the vicinity to vast vegetated areas, high biodiversity (due to nearby lakes and river vegetation), no traffic, and no agricultural activities could be associated with the lowest pollution identified in this case.

### 3.2. Relationship between Heavy Trace Elements

**Figure 1:**
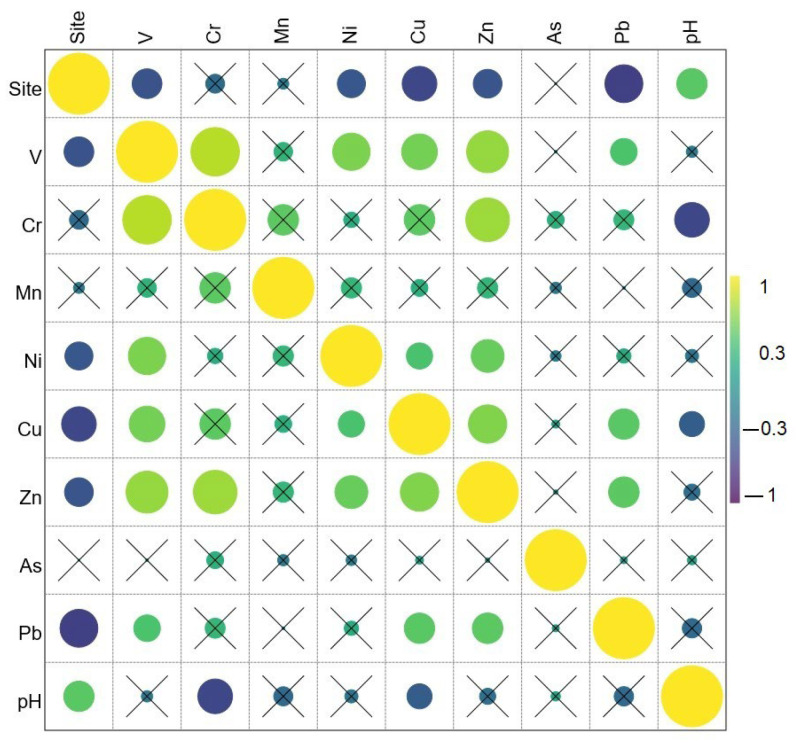
Kendall tau correlogram showing relationship between variables; yellow—perfect positive *τ* correlation, dark purple—perfect negative *τ* correlation; crossed (“×”) not significant at *p* < 0.05; size of the bubble is proportional to the coefficient value.

The relationship between the screened elements is presented in Figure 1. The strongest monotonous relationship was found between V and Cr (*τ* = 0.794). Zn was significantly positively correlated with Cr (*τ* = 0.714), V (*τ* = 0.687), Cu (*τ* = 0.627), and Ni (*τ* = 0.537). Pb also correlated positively with Zn (*τ* = 0.500), Cu (*τ* = 0.497), and V (*τ* = 0.436) (Figure 1). Positive significant correlations between certain elements associated with higher than background levels at some locations could indicate a common source of pollution with those elements. Therefore, for the landscaping architect, this would indicate that choosing plants able to tolerate or remediate both of those correlated heavy metals would be the best option.

## 4. Discussion

This study screened the presence of some of the most common heavy metals, which present elevated levels in urban environments across Europe [10], in six playgrounds in Cluj-Napoca, Romania, using the XRF technique. The results indicate that this method is effective in identifying the eight targeted heavy metals (V, Cr, Mn, Ni, Cu, Zn, As, and Pb), and that their average levels per site are within safe intervals as defined by the current national legislation [33]. A review of heavy metals in urban soil across Europe showed that the most common metals that exceed national safety thresholds are Pb, Zn, Cu, Cr, and Ni [10].

Among the screened elements, As exceeded worldwide average values at all studied sites. Arsenic was introduced into the environment by burning As-rich coal and by the utilization of arsenical compounds in agriculture, particularly pesticides in the form of simple inorganic As salts. Wood preservatives (e.g., chromated copper arsenate) still account for 30% of the world arsenic market [41], and were shown to contribute to soil pollution with Cu [42]. HM pollution of urban soil in Europe showed that elevated levels of the most common five heavy metals were 22% of anthropogenic enrichment (such as traffic and industry) while 44% were of geogenic enrichment [10]. In Cluj-Napoca, the main source of metal pollution is related to traffic, urban run-off, residential heating, and municipal landfill [19]. The proximity to pollution sources can be a cause of increased soil pollution in the urban environment. This takes place by suspension and accumulation in the atmosphere during dry weather, transport with air masses, deposition on vegetation and surfaces, and eventually wash-off into the ground with rainwater [43]. In this way, heavy metals such as those screened in this study can end up in the soil. Construction materials used in roads and pathways can also be a source of HM, as shown for asphalt, pavement, or crushed stone via stormwater run-off, which had measurable levels of Cu, Pb, and Zn [44]. In addition, concrete pavement can leach Cr, since it is found in Portland cement [43]. Therefore, the type of materials used for pathways should be carefully chosen to eliminate/limit the use of those with a slow release of HMs. Among the soil parameters, pH influences both the solubility [45] and mobility [46,47] of heavy metals. In this regard, a higher acidity of the soil increases the mobility of metal ions [48,49]. The natural soil components responsible for metal elements sorption are soil humic substances, phyllosilicates, carbonates, and variable charge minerals, as well as soil microorganisms, but heavy metals compete for sorption sites onto soil components [50]. Therefore, manipulating and maintaining certain soil/substrate quality can contribute to reducing the exposure risk. Such approaches could seek to leverage the soil particularities, which could mitigate heavy metals through their buffering effects by ensuring neuter pH, high humus and carbonate content, the predominance of loamy and loamy-clay texture, and low salt content [51]. The neutralization/correction of acidic soil can be achieved using CaCO_3_, bentonite, and volcanic tuff to enhance the buffering capacity of the soils [49].

Besides corrections to enhance the soil buffering capacity, substantial improvement in urban soils that are polluted can be achieved in a variety of ways: importing soil and applying soils amendments (particularly biosolids) [52], mineral materials [53], soil removal and soil isolation, replacement of contaminated soil, electrokinetic remediation, soil leaching (washing), adsorption, washing and compounding, heat treatment, physical solidification, chemical improvers, chemical curing lamp remediation, and bioremediation (including phytoremediation and microbial remediation) [54]. The scale of these interventions can be decided based on the level of contamination identified and its threat.

Out of these possible interventions, phytoremediation relies on plants to mitigate heavy metals. This method could be paired with the application of beneficial microorganisms such as arbuscular mycorrhizal fungi for joined phytoremediation–mycoremediation [55]. The phytoremediation method has received increasing attention because it involves lesser costs in scenarios of moderate polluted soils [56]. This green technology relies on several mechanisms: (1) phytoextraction (through which metals are extracted by the plant and accumulate in the harvestable plant parts); (2) rhizofiltration (heavy metals are sequestrated and precipitate at the level of underground plant part); (3) phytostabilization (decreasing the environmental burden of pollutants by stabilizing them through root sorption and chemical fixation, which reduces the migration of pollutants); and (4) phytovolatilization (mobilizing pollutants from soil and once these are converted into less toxic gaseous forms, they are released into the atmosphere) [57]. While some of these mechanisms are merely an immobilization of heavy metal pollutants without actually removing them, they have relevance in reducing exposure risk. Out of all these, the use of hyperaccumulator plants, able to extract and accumulate the pollutants in the aboveground parts in significant amounts, is more valuable. Repeated harvesting and disposal of biomass can actually reduce the levels of given contaminants in the soil. Although the harvested contaminated biomass raises further environmental concerns, some responsible disposal or up-cycling options exist [58]. There are known to be about 400 metal hyperaccumator species of plants, belonging to 22 botanic families. The Brassicaceae family comprises no less than 87 such species, with the broadest ranges of metal accumulation properties [59]. Ornamental plant species with heavy metal phytoremediation capacity have to be integrated based on landscaping principles. Thus, herbaceous cover providing dense root mats and comprised of species that can prevent vertical lift-up of dust with heavy metals could be complemented by woody plant species that can extract and accumulate heavy metals in their biomass. Due to specificity, choosing the right plant species for specific heavy metal conditions the success of the intervention [60]. The plant species has to also be chosen based on environmental requirements, such as soil pH, climatic conditions, rainfall, and light availability. Candidate plant species can be selected from available databases such as the one by Nelson et al. [61] and Phytorem [62]. The plant species integrated into the landscape can also be used for biomonitoring [63].

Suggestions for landscaping on Zn and As-enriched soil (such as those in this study) include grass species such *Cynodon dactylon*, to ensure a dense ground cover and woody species such as *Pinus densiflora*. The species *Robinia pseudoacacia* and *Amorphoa fruticosa* are tolerant to As and Pb, alongside several *Salix* species (*S. dasyclados*, *S. triandra*, *S. fragilis*, and *S. schwerinii*) that can extract Zn from soil, and could be considered in a planting plan. Herbaceous cover of *Trifolium fragiferum* and *Hibiscus cannabinus* can be used for Pb phytoextraction [61]. There are many more options available to landscaping specialists depending on the design schemes and identity of target HMs.

Comparing the results in the current study with the levels reported in previously published works, V levels in the top soil of studied playgrounds from Cluj-Napoca are lower than those reported in Yerevan kindergarten soil, but similar to the mean level measured in playgrounds from Bratislava (Table 5). The range of Zn levels was lower than the mean levels reported in Belgrade (Serbia) or Hong Kong (China). The Pb levels were similar to the ranges measured in coastal municipalities of Montenegro, but the mean of the six sites was higher than the mean reported in Çanakkale (Turkey) and Uppsala (Sweden) (Table 5).

A previous study showed that soil samples in an urban area of Cluj-Napoca had on average Cu 41 mg/kg, Pb 53 mg/kg, Zn 125 mg/kg, Cr 46 mg/kg, and Ni 47.5 mg/kg [19], compared to these, the values in this study were lower. A study conducted in the country’s capital (Bucharest) showed that three major urban parks had elevated Zn, Pb, and Cu levels, and although heavy metal concentration was heterogenous, the overall proximity to intense trafficways was proposed as a source of pollution [74]. A study conducted in Poland across playgrounds and sport facilities in Warsaw and Bydgoszcz, which are frequently used by children and youth, proposed that although classified as uncontaminated (with only Pb and Zn exceeding background levels), lowering permissible limits in soils and monitoring their levels in recreational areas could help to decrease exposure risk for children [69]. An extensive study from Hungary screened 96 public parks and 89 playgrounds across three cities: Budapest, Szeged, and Gyula, for heavy metals. Out of the sites assessed, 36 exceeded the threshold values as stipulated in the respective national legislation [51], indicating the widespread issue of heavy metals in urban outdoor areas usually associated with healthy activities, within large cities. A study conducted in Greece screened for heavy metals in the soils from some children’s playgrounds across Athens and identified that high enrichment cases with heavy metals were site-specific. The increased levels of Cr, Zn, Mn, Pb, and Ni were attributed to recent pollution events, through atmospheric deposition [67]. Screening of heavy metals across playgrounds from public parks and kindergartens in seven coastal municipalities of Montenegro revealed higher than background levels for Pb and Cd. Additionally, while Cr presence was due to natural sources, Pb, Cd, Cu, and Zn levels were associated with anthropogenic sources [68]. A study of dust from children’s playgrounds in Hong Kong indicated that metal content was significantly correlated with traffic volume. In addition, Pb and Zn were correlated, suggesting a common source [66]. Similarly, in this study, increased levels of both Zn and Pb were found at the two sites that are near intense traffic driveways.

In this study, the soil sample analyses with the portable XRF were performed in the laboratory. One shall consider that while the XRF method brings many advantages, there are also some potential drawbacks related to the factors that can influence the accuracy of the results. It has been shown that, with careful consideration of such factors, XRF measurements can be highly accurate [75]. Still, for making this method widely useful for landscaping specialists, some optimized protocols are required. The field soils can often be moist, which decreases the accuracy of the measurements and, therefore, soils might require drying. Nevertheless, the need for a minimal preparation step for the samples should not be a hurdle. The equipment used here was able to perform relevant readings for the selected HMs from this study that were compared to legislation thresholds. However, some other hazardous HMs have low admissible/normal thresholds in soil (e.g., Cd, Hg), therefore the equipment used should be able to detect those alert and intervention levels from legislation (therefore the limit of detection should be lower than the intervals defined by legislation), otherwise other, more sensitive approaches would be needed.

In some countries, such as the Netherlands, there are protocols for the XRF measurements of soil that are available for local environmental restoration consultants [76], while guidelines to monitor hazardous HMs such as Pb in playgrounds have been set in place [77]. Still, this is not yet mainstream and such examples could pave the way for other countries to follow. At the current date, for the region of study, there are no binding policies in place for playgrounds in regard to HMs monitoring, and HMs measurements remain voluntary for landscaping specialists. The greatest benefit of implementing preliminary HMs screening would be in customizing vegetation options based on principles of phytoremediation, which would be at hand for specialists designing/re-designing these spaces.

Vegetation presence in public playgrounds has a highly positive influence on children’s well-being, particularly when these include trees and shrubs, since these can protect children from unhealthy amounts of solar radiation [78]. Another argument in favor of strongly considering diverse vegetation in children’s playgrounds is that vegetation from urban green spaces contributes to air purification and the reduction in particulate matter load that is suspended in breathable air [79], which is one of the leading pollution issues in cities [20]. Therefore, we propose that all three levels of vegetation should be considered when designing playgrounds: herbaceous cover, low shrubby vegetation, and trees. As such, XRF measurements could be optimized with specific protocols for assisting plant species selection and vegetation composition schemes.

Because urban parks and public children’s playgrounds are associated with health-promoting activities and functions, one can infer their quality is worth more attention in order to ensure that they have the expected beneficial role [80]. A better understanding of factors that contribute to the degradation of urban soil quality and identifying available options to mitigate these undesired processes would help to design safer playgrounds. In addition, the constant monitoring of heavy metals in recreative spaces, particularly destined for children, should become mainstream in order to ensure prompt interventions when needed. Particularly, there is a stringent need for standardized protocols and operation procedures addressed to different categories of specialists, and these should be adapted to their specific types of activities. Since heavy metals have become closely linked to anthropogenic space, a multidirectional mitigation approach should be considered, since these are becoming widely common pollutants in cities. Diverse professional segments at local level could contribute together to preventing or tackling this HM contamination problem in cities, therefore optimized protocols and procedures should be specifically addressed to them.

## 5. Conclusions

Landscaping specialists are the first line of action in designing playgrounds and there is a necessity to optimize approaches to help them respond in a timely way to current HM pollution challenges. Given the vulnerability of children to the negative impact of heavy metal pollution, routine HM monitoring of children’s playgrounds should become mainstream. To make this possible, XRF and optimized protocols for landscape consultants should be made available at local level.

We studied the presence of eight common heavy metals (V, Mn, Ni, Pb, Cu, Zn, Cr, and As) using the XRF method across six children’s playgrounds of different typologies in Cluj-Napoca, Romania. These HMs are among the ones reportedly presenting elevated levels across urban ecosystems Europe-wide. It was determined that the XRF method can provide quick orientation in improving and designing these spaces, particularly with regard to vegetation options. The main drawback remains related to the careful consideration of factors that can affect the accuracy of the readings.

Average levels of all eight heavy metals identified by portable XRF were below intervention thresholds at all sites, and seven out of eight were also below alert level (with the exception of As at one site). The pollution load index showed that two playgrounds had no pollution (*PLI* = 0.89–0.99), and three of them showed only baseline pollution with incipient deterioration in soil quality (*PLI* = 1.01–1.51). The highest *PLI* depending on the location was due to Zn, Pb, As, and Mn; therefore, their levels should be monitored, and the vegetation chosen for these sites should consider species with phytoremediation capacity for these elements. An examination of the relationship between parameters using the Kendall tau coefficient showed that Zn displayed a significantly positive monotonic correlation with five of the heavy metals identified (Cr, V, Cu, Ni, and Pb), suggesting a common source. It was observed that the playgrounds least threatened by pollution were located away from intense car traffic and neighbored vast vegetated areas, in consensus with previous reports.

Because publicly accessible green space per inhabitant in Cluj-Napoca is below the European average, more green spaces are needed across residential areas. The incorporation of playgrounds within larger vegetated parks, with plant species carefully chosen based on previsioning pollution sources and trends, might ensure the increased quality of these spaces.

## Figures and Tables

**Table 1:** Locations of children’s playgrounds studied in Cluj-Napoca.

Site Acronym	Location and Elevation	Vegetation	Pathways Type
CJ-AM1	Andrei Mureșanu site 1, 371 m	Lawn, hedge, trees	Concrete pavement
	46°45′39.09″ N 23°36′12.86″ E		
CJ-AM2	Andrei Mureșanu site 2, 369 m	Lawn, hedge, trees	Asphalt, rubber pavement
	46°45′42.39″ N 23°36′22.63″ E		
CJ-BZ1	Bună Ziua district site 1, 443 m	Lawn	Gravel, rubber pavement
	46°45′7.04″ N 23°36′13.18″ E		
CJ-BZ2	Bună Ziua district site 2, 459 m	Lawn	-
	46°44′56.29″ N 23°36′2.06″ E		
CJ-CB	Colonia Borhanci, 352 m	Lawn	-
	46°44′56.58″ N 23°38′22.68″ E		
CJ-G	Gheogheni district, 333 m	Lawn, shrubs, trees	Concrete and rubber pavement
	46°46′5.45″ N 23°38′0.82″ E		

**Table 2:** Soil heavy metals in some public children’s playgrounds and influence of the site location (Cluj-Napoca, 2022) (*n* = 18).

Parameter	Descriptive Statistics							Analysis of Variance		
	Average (mg/kg)	Median (mg/kg)	±SD	Skew	Max.	Min.	CV	Test Value	*p*	Sign.
V	69.78	74.00	20.14	−0.50	100	31	28.86	*F* = 2.51	0.0891	ns
Cr	35.00	31.00	14.14	1.27	63	20	40.40	*F* = 0.51	0.7662	ns
Mn	475.11	362.00	466.29	3.31	2280	108	98.14	*χ^2^* = 5.13	0.4005	ns
Ni	33.50	31.50	12.92	0.48	62	14	38.57	*F* = 2.06	0.1417	ns
Cu	26.75	22.00	12.71	0.97	53	15	47.51	*χ^2^* = 10.61	0.0597	ns
Zn	106.94	86.00	51.86	1.21	235	58	48.49	*χ^2^* = 13.18	0.0218	*
As	13.92	13.00	4.14	1.95	25	10	44.85	*χ^2^ =* 8.05	0.1536	ns
Pb	32.13	35.00	14.41	0.80	67	14	29.74	*F* = 3.33	0.0561	ns

Note: maximum and minimum (Max., Min.); coefficient of variation (CV); *F* according to parametric one-way ANOVA test and *χ^2^* according to non-parametric Kruskal–Wallis test; significance (Sign.) *p* > 0.05 (ns), *p* < 0.05 (*).

**Table 3:** Levels (mg/kg) of heavy metals and pH for the top soil from six public children’s playgrounds (*n* = 18) of Cluj-Napoca, Romania (2022).

Criteria	Parameter	V	Cr	Mn	Ni	Cu	Zn	As	Pb	pH
CJ-AM1	Mean	84.00	35.67	581.00	44.00	43.00	145.67 ^a,b^	13.00	40.00	6.80
	±SE	2.89	6.49	116.52	2.52	2.25	7.36	1.00	1.53	0.06
CJ-AM2	Mean	92.33	49.50	447.67	44.33	37.33	183.33 ^a^	13.67	47.00	6.90
	±SE	5.36	13.50	102.92	11.05	8.95	33.34	0.88	11.85	0.06
CJ-BZ1	Mean	66.67	20.00	297.67	30.33	16.67	76.00 ^b^	21.50	27.00	7.07
	±SE	6.33	-	54.81	1.86	0.67	5.03	3.50	8.00	0.03
CJ-BZ2	Mean	54.67	29.00	947.00	20.67	19.50	78.67 ^b^	13.00	16.00	7.03
	±SE	16.74	-	631.10	4.41	2.50	11.26	-	2.00	0.03
CJ-CB	Mean	65.67	25.00	254.67	34.00	22.00	94.00 ^a,b^	11.33	33.67	7.03
	±SE	8.82	-	138.25	9.64	5.00	20.00	0.88	1.33	0.03
CJ-G	Mean	55.33	<LOD	322.67	27.67	18.00	59.67 ^c^	10.00	17.00	6.87
	±SE	11.26	-	49.82	3.18	2.08	0.88	-	3.00	0.07
XRF	LOD	15	15	50	10	10	50	10	10	-
Positive number of samples (no.)	no. ≥ LOD	18	8	18	18	16	17	12	15	-
National legislation [33] thresholds for sensitive use	Normal	50	30	900	20	20	100	5	20	-
	Alert	100	100	1500	75	100	300	15	50	-
	Intervention	200	300	2500	150	200	600	25	100	-
Romanian soils average, ICPA [40]	Agricultural top soil	n/a	n/a	513.14	34.49	26.07	87.34	n/a	21.3	-
European Soil Database, Utermann et al. [34]	Geochemical background MAT11LU-6	n/a	47	n/a	29	20	60	n/a	17	-
Worldwide average Kabata-Pendias et al. [35]	Silty and loamy soils	76	51	525	26	23	60	8.4	28	-

Note: ±SE—standard error of mean; n/a—unspecified; LOD = limit of detection; different letters indicate significant differences between means, Dunn test (*p* < 0.05); ICPA—National Research-Development Institute for Pedology, Agrochemistry and Environmental Protection in Bucharest.

**Table 4:** Ranking pollution index values for the measured heavy metals in the soil of six public children’s playgrounds in Cluj-Napoca (2022).

Parameters		Sites					
		CJ-AM1	CJ-AM2	CJ-BZ1	CJ-BZ2	CJ-CB	CJ-G
*PI*	V	1.11 (2)	1.21 (2)	0.88 (1)	0.72 (1)	0.86 (1)	0.73 (1)
	Cr	0.76 (1)	1.05 (2)	0.43 (1)	0.62 (1)	0.53 (1)	<LOD
	Mn	1.11 (2)	0.85 (1)	0.57 (1)	1.80 (2)	0.49 (1)	0.61 (1)
	Ni	1.52 (2)	1.53 (2)	1.04 (2)	0.71 (1)	1.17 (2)	0.96 (1)
	Cu	2.15 (3)	1.87 (2)	0.84 (1)	0.98 (1)	1.10 (2)	0.90 (1)
	Zn	2.43 (3)	3.06 (4)	1.27 (2)	1.31 (2)	1.57 (2)	1.00 (1)
	As	1.55 (2)	1.63 (2)	2.56 (3)	1.55 (2)	1.35 (2)	1.19 (2)
	Pb	2.35 (3)	2.76 (3)	1.59 (2)	0.94 (1)	1.98 (2)	1.00 (1)
*PLI*		1.51	1.60	0.99	1.01	1.02	0.89
		II	II	I	II	II	I

Note: numbers in parentheses indicate the class of pollution threat: class (1), *PI* < 1 = soil pollution absent; class (2), 1 < *PI* < 2 = low; class (3), 2 < *PI* < 3 = moderate; class (4), 3 < *PI* < 5 = strong; rank I 0 < *PLI* ≤ 1 = no pollution status; rank II 1 < *PLI* ≤ 2 = low/baseline pollution with slight deterioration in soil quality.

**Table 5:** Levels of heavy metals (mg/kg) in urban top soil from children’s playgrounds.

Location	Sites	Methods	Heavy Metals (mg/kg)	Sources
Armenia (Yerevan)	Kindergarten soil	XRF	As 0.69, Cr 66.4, Cu 57.9, Mn 830, Ni 31.4, Pb 2.4, V 98.7, Zn 195	[64]
Chile (Biobio region cities)	Playgrounds	ICP-MS	As 19.51, Cr 32.90, Cu 31.51, Ni 23.76, Pb 17.59, Zn 63.69,	[65]
China (Hong Kong)	Playgrounds	AAS	Cu 28.4, Pb 195, Zn 237,	[66]
Greece (Athens)	Playgrounds	AAS	Cr 79.9, Cu 43.4, Mn 311.6, Ni 81.5, Pb 110.3, Zn 174.3	[67]
Montenegro (coastal municipalities)	Public parks and kindergartens	GF-AAS, ICP-OES	Cr 5.55–32.51, Cu 26.11–124.06, Pb 2.86–33.30, Zn 14.02–67.88,	[68]
Poland (Warsaw, Bydgoszcz)	Public playgrounds, sport facilities	ICP-MS, EDXR	Cu 13–57.4, Pb 8.7–167, Zn 16–325	[69]
Serbia (Belgrade)	Green areas near elementary schools, kindergartens	ICP-OES	Ni 46.79, Zn 223.11	[70]
Slovakia (Bratislava)	Playgrounds	ICP-MS	As 8.30, Cr 44.1, Cu 40.9, Mn 609, Ni 25.6, Pb 32.3, V 64.7, Zn 109	[71]
Sweden (Uppsala)	Public/daycare playgrounds	ICP-AES, ICP/MD-DRC	As 3.4, Cr 32, Cu 25, Mn 494, Ni 19, Pb 26, Zn 84	[72]
Turkey (Çanakkale)	Playgrounds	ICP-OES	Cr 21, Cu 28, Mn 475, Ni 21, Pb 18, Zn 58	[73]

Note: Atomic Absorption Spectroscopy (AAS), Graphite Furnace (GF-), Dynamic Reaction Cell (-DRC), Inductively Coupled Plasma Mass Spectrometry (ICP-MS), Inductively Coupled Plasma Atomic Emission Spectroscopy (ICP-AES), Inductively Coupled Plasma Atomic Emission Spectroscopy (ICP-OES), Energy Dispersive X-ray Fluorescence (EDXR), X-ray Fluorescence (XRF).

## Data Availability

Data available in the main text of the paper.
